# Cognitive impairments predict the behavioral and psychological symptoms of dementia

**DOI:** 10.3389/fneur.2023.1194917

**Published:** 2023-07-20

**Authors:** Solaphat Hemrungrojn, Sookjaroen Tangwongchai, Thammanard Charernboon, Muthita Phanasathit, Pisit Chaipresertsud, Pacharaporn Maleevach, Yuttachai Likitjaroen, Kammant Phanthumchinda, Ratiya Assawatinna, Arisara Amrapala, Michael Maes

**Affiliations:** ^1^Department of Psychiatry, Faculty of Medicine, Chulalongkorn University and King Chulalongkorn Memorial Hospital, the Thai Red Cross Society, Bangkok, Thailand; ^2^Cognitive Fitness and Biopsychiatry Technology Research Unit, Faculty of Medicine, Chulalongkorn University, Bangkok, Thailand; ^3^Cognitive Impairment and Dementia Research Unit, Faculty of Medicine, Chulalongkorn University, Bangkok, Thailand; ^4^Department of Clinical Epidemiology, Faculty of Medicine, Thammasat University, Pathumthani, Thailand; ^5^Department of Psychiatry, Faculty of Medicine, Thammasat University, Pathumthani, Thailand; ^6^Chao Phraya Abhaibhubejhr Hospital, Prachinburi, Thailand; ^7^Angthong Hospital, Ang Thong, Thailand; ^8^Division of Neurology, Department of Medicine, Faculty of Medicine, Chulalongkorn University, Bangkok, Thailand; ^9^Department of Psychiatry, Medical University of Plovdiv and Technological Center for Emergency Medicine, Plovdiv, Bulgaria; ^10^Kyung Hee University, Seoul, Republic of Korea; ^11^Mental Health Center, University of Electronic Science and Technology of China, Chengdu, China; ^12^Research Institute, Medical University of Plovdiv, Plovdiv, Bulgaria

**Keywords:** cognitive dysfunction, behavioral dysfunction, Alzheimer’s disease, psychiatry, neuropsychiatric symptoms, memory

## Abstract

**Introduction:**

The purpose of this study was to (1) validate the Thai version of the Neuropsychiatric Inventory Questionnaire (NPI-Q) as a screening tool for behavioral and psychological symptoms of dementia (BPSD), and (2) examine the relationship between cognitive performance and BPSD in an elderly population with amnestic mild cognitive impairment (aMCI) and dementia of Alzheimer’s type (DAT).

**Methods:**

One hundred and twenty participants, comprising 80 aMCI and 40 DAT patients, and their respective caregivers were included in the study. Participants completed the NPI-Q and the Neuropsychiatric Inventory (NPI) within 2 weeks of each other and cognitive performance was primarily assessed using the Montreal Cognitive Assessment (MoCA).

**Results:**

The Thai NPI-Q had good validity and reliability. Pure exploratory bifactor analysis revealed that a general factor and a single-group factor (with high loadings on delusions, hallucinations, apathy, and appetite) underpinned the NPI-Q domains. Significant negative correlations between the MoCA total score and the general and single-group NPI-Q scores were found in all subjects (aMCI + DAT combined) and DAT alone, but not in aMCI. Cluster analysis allocated subjects with BPSD (10% of aMCI and 50% of DAT participants) into a distinct “DAT + BPSD” class.

**Conclusion:**

The NPI-Q is an appropriate instrument for assessing BPSD and the total score is largely predicted by cognitive deficits. It is plausible that aMCI subjects with severe NPI-Q symptoms (10% of our sample) may have a poorer prognosis and constitute a subgroup of aMCI patients who will likely convert into probable dementia.

## 1. Introduction

Presently, Thailand’s total population has surpassed 70 million and has been an aging society since the early 2000s, with over 10% of the population aged 60 and above (1). In 2015, it was estimated that approximately 600,000 elderly Thai people were living with dementia, mainly caused by dementia of Alzheimer’s type (DAT) and vascular dementia (2). These numbers are projected to increase to 1 million by the year 2030 (3).

The aging process is often accompanied by amnestic mild cognitive impairment (aMCI), an intermediate stage between natural aging and probable DAT (4). It is estimated that those with aMCI in community settings and specialty clinics develop DAT at an annual conversion rate of 3–10% and 10–15%, respectively, while the remaining either stabilize or convert back to a normal aging process (5).

In addition to cognitive deficits, aMCI and DAT are associated with neuropsychiatric symptoms. These neuropsychiatric symptoms are collectively known as behavioral and psychological symptoms of dementia (BPSD) and encompass the following domains: delusions, hallucinations, agitation/aggression, depression/dysphoria, anxiety, euphoria, apathy/indifference, disinhibition, irritability/lability, aberrant motor activity, nighttime behavioral disturbances, and appetite and eating abnormalities (6). Currently, the most widely used instrument to measure BPSD is the neuropsychiatric inventory (NPI) (7, 8), which also exists in a questionnaire form (NPI-Q) (9). Although BPSD are not a hallmark of aMCI as defined by its diagnostic criteria and the label contains the word ‘dementia’, BPSD is present in most individuals throughout dementia progression, including the earlier stages of cognitive impairment (10). Up to 90% of dementia patients exhibit BPSD at any given point over the course of their illness (11). Most aMCI and DAT individuals seem to have at least one persistent BPSD symptom (12, 13), whereby symptoms are generally more prevalent and severe in DAT than in aMCI (14). In the Thai aMCI population, the most common BPSD are irritability, nighttime disturbances, and anxiety (15). On the other hand, the most prevalent BPSD found in DAT are apathy, irritability, sleep disorders, agitation, and aberrant motor behavior (13).

The detection and characterization of BPSD are essential, as these symptoms may predict a faster cognitive decline (16) and further reduce the individual’s quality of life (17). Studies have shown that there is an inverse relationship between BPSD (severity and frequency) and cognitive ability (9, 16, 18, 19). However, the existing studies were conducted in the West as well as in East Asia, highlighting the concern that the previous findings may not apply to other cultures or countries, including Thailand, and there is a lack of exploration on the relationship between BPSD and cognitive performance in unreported countries. The local context is important as cultural differences may lead to variability in symptom and cognitive manifestations, which could ultimately affect patient treatment and management.

The purpose of this study was to (1) validate the Thai version of the NPI-Q as an adequate instrument to screen for BPSD, and (2) examine the relationship between cognitive performance and BPSD in an elderly population with aMCI and DAT. Cognitive performance was assessed using the Thai version of the Montreal Cognitive Assessment (MoCA), an excellent assessment tool for neurocognitive impairment (20), and the Thai Mini-Mental State Examination (TMSE). It was hypothesized that the Thai NPI-Q will have good validity and reliability and that lower cognitive performance would be associated with higher BPSD severity.

## 2. Materials and methods

### 2.1. Translation and cross-cultural adaptation of the NPI-Q

The NPI-Q was translated into Thai with permission from Dr. Jeffery Cummings. Forward translation into Thai was performed by two independent, bilingual Thai-English translators. Literal translation was avoided, and simpler, context-relevant wordings were used to suit the Thai elderly population. Afterwards, the translation from each individual translator was compared and discussed, and a first draft of the translated version was produced once a consensus was reached.

Back translations were compared to the initial English NPI-Q version by a professional panel consisting of two psychiatrists, one geriatric psychiatrist, and one neuropsychologist. Content validity of the draft translation was assessed by a panel of three experts who were not involved in the initial translation (two neuropsychiatrists and one researcher with experience in the development and validation of instruments). To determine content validity, these experts were asked to score each item on the questionnaire as (1) not, (2) somewhat, (3) very, or (4) extremely relevant. The relevance content validity index (CVI) for individual items was calculated as the number of judges who voted “very” or “extremely relevant”/total number of experts recruited. Items with a CVI of 0.8 or above were retained. Accordingly, no items were discarded. A pre-final version of the Thai translation was drafted and administered in a pilot study to 10 caregivers of dementia patients to assess clarity and understanding of test items. Then, face-to-face caregiver interviews were conducted to determine if they felt any difficulties or ambiguity in responding to the test items. None of the caregivers reported any problems in understanding the scoring scale, thus, no further modifications were made, and the Thai NPI-Q was finalized.

### 2.2. Participants

A total of 120 participants and their respective caregivers were recruited for the study: 80 aMCI and 40 patients with DAT from three outpatient departments (OPDs) in King Chulalongkorn Memorial Hospital.

The inclusion criteria for the patients were to be 50 years or older and living in the same catchment area (Bangkok, Thailand). The study was limited to people 50 years or older as past research on BPSD in aMCI/DAT predominantly focused on this age range (21, 22). All patients with aMCI complained of subjective memory problems and were diagnosed using Petersen’s Criteria (4), which include (1) subjective memory problems, (2) abnormal memory function for age, (3) normal general cognitive function, (4) normal activities of daily living, and (5) absence of dementia. Subjective memory complaints were assessed using the question “do you feel as if your memory is deteriorating?” (23). aMCI participants were included if they had a Thai Clinical Dementia Rating (CDR) (24) score of 0.5 and a memory subdomain score of 0.5. DAT was diagnosed using the National Institute of Neurological and Communicative Disorders and Stroke/AD criteria and the International Statistical Classification of Diseases and Related Health Problems, Tenth Revision, (ICD-10) and a Thai CDR of 1.

Patients who were terminally ill, had severe hearing and visual deficits, had major axis I psychiatric disorders (including major depression, bipolar disorder, schizophrenia, autism, and delirium) or were uncooperative were excluded in the study. We also excluded subjects who were undergoing treatments/interventions (both pharmaceutical and non-pharmaceutical) for dementia or BPSD, as well as those with comorbid medical illness including stroke, multiple sclerosis, Parkinson’s disease, encephalitis, traumatic brain injury, epilepsy, meningitis, COPD, rheumatoid arthritis, inflammatory bowel disease, stroke, and cardiovascular disorder.

As the NPI and NPI-Q are informant-based assessments, the caregivers of the patients were eligible for participation in this study if they (1) could read, write, speak, and understand Thai language well, and (2) were family members/hired caregivers involved in the patient’s daily care who were fully aware of their behavior, or relatives who visited the patients more than twice per week.

### 2.3. Study measurements

The NPI was first developed by Kaufer et al. (8), and is a retrospective, informant-based interview that uses scripted questions to assesses the presence of 12 neuropsychiatric symptoms: delusions, hallucinations, agitation/aggression, depression/dysphoria, anxiety, euphoria, apathy/indifference, disinhibition, irritability/lability, aberrant motor activity, nighttime behavioral disturbances, and appetite and eating abnormalities (6). Each neuropsychiatric symptom experienced by the patient within the last 4 weeks is rated by the patient’s caregiver in terms of frequency. A composite score is calculated for each domain by multiplying the frequency by severity. The total NPI score represents the sum of all individual composite scores giving a value from 0 to 144. The total distress score represents the sum of the associated caregiver distress score of each symptom and ranges from 0 to 60. The NPI is administered in the form of a structured interview that takes approximately 15–30 min to administer depending on dementia severity and requires a trained assessor (25). Its iterations have demonstrated great validity and reliability over time and is now used worldwide with at least 75 translations into other languages and dialects, including Thai (26).

The NPI-Q is an informant-based questionnaire by Monastero et al. (9) that is a brief questionnaire form of the standard NPI test evaluating the same 12-symptom domains. It can be completed within 5–10 min. Each domain is assessed by a screening question that determines presence (0: no or 1: yes) and severity (1/3: mild; 2/3: moderate; and 3/3: severe) of the symptoms present within the last 4 weeks. The total NPI-Q severity score represents the sum of individual symptom scores and ranges from 0 to 36. The caregiver’s distress is also rated for each neuropsychiatric symptom domain on a six Likert scale from 0: “not distressing at all” to 5: “extremely distressing.” The total distress score represents the sum of the associated caregiver distress score of each symptom and ranges from 0 to 60. The NPI-Q authors demonstrated that the scale has adequate test-retest reliability and that the interscale correlation between the NPI-Q and the NPI was sufficient (9).

The MoCA evaluates 6 cognitive domains (20): (1) executive function; (2) visuospatial; (3) short-term memory; (4) language; (5) attention, concentration, and working memory; and (6) temporal and spatial orientation. A score of 17–24 represents aMCI, while a score of ≤16 represents DAT. We employed the raw MoCA total score (sum of all items), the adapted MoCA score (score + 1 when years of education is <6 years), as well as all MoCA subdomain scores in our analyses (20).

The Thai Mini-Mental State Examination (TMSE) is an adaptation of the MMSE by Folstein et al. (27) and assesses severity of cognitive impairments in six domains: (1) orientation; (2) registration; (3) attention; (4) calculation; (5) language; and (6) recall (28). Scores of ≤23 indicate dementia.

### 2.4. Procedure

Recruitment flyers were posted in three OPDs inviting people with the desired characteristics to volunteer. Interested volunteers were given information on the research procedures and then screened using the inclusion and exclusion criteria. Those who met the criteria were invited to participate in the study. After obtaining written consent, an experienced research assistant proceeded with data collection. The patient’s socio-demographic characteristics were recorded, specifically age, gender, marital status, living condition, and level of education. Education level was assessed as an ordinal scale, namely: 1 – no education, 2 – below primary 6, 3 – primary school, 4 – middle school, 5 – secondary school, 6 – diploma, and 7 – bachelor’s degree or higher. Clinical data of the patients, including medical and medication history, was acquired from existing medical charts. Subsequently, the NPI-Q was given to caregivers to complete. Within 2 weeks after completing the NPI-Q, the caregivers were assessed using the standard NPI via scripted interview—a different research assistant conducted the interview to avoid researcher bias. Both research assistants were blinded to the results of the other test.

To assess test-retest reliability, 20 caregivers were randomly selected to complete the NPI-Q test a second time within 1 week of the first test. A one-week duration between test-retest was chosen as the retest should be administered within 4 weeks; otherwise, the patient’s clinical picture may be altered, consequently compromising the validity of the test-retest assessment. Data collection and administration of the second test was carried out by the same research assistant as the first test. Moreover, to assess inter-rater reliability, another additional 20 caregivers were randomly selected to complete the NPI-Q a second time within 1 week of the first test. For this second test round, a new research assistant (blinded to the result of their first test) administered the NPI-Q and collected the data.

### 2.5. Statistical analyses

#### 2.5.1. Demographics

Demographics and clinical data of patients were summarized using descriptive statistics. Analysis of variance was used to assess differences in continuous variables between groups, while contingency analysis (χ^2^ test) or the Fisher-Freeman-Halton Exact test were employed to check associations between categorical data.

#### 2.5.2. NPI-Q validity/reliability

The intra-class correlation coefficient (two-way random model) was used to check test-retest reliability. The internal consistency of the NPI-Q test was analyzed using Cronbach’s coefficient alpha. Concurrent validity was tested by examining Spearman’s rank order correlations between the NPI-Q and MoCA scores in study groups (we expect significant negative associations), while discriminant validity was assessed by computing these associations in aMCI (no significant associations are expected). This type of correlation analysis was used because the NPI data are not normally distributed.

#### 2.5.3. Psychometric properties of the NPI-Q

Exploratory Factor Analysis (EFA) was used to study the component structure of the NPI-Q and to determine whether a general factor could be extracted from the NPI-Q items. The Kaiser-Meyer-Olkin (KMO) metric was used to assess whether the data was suited for factor analysis.

#### 2.5.4. Associations between cognitive performance and BPSD

Machine learning techniques were employed in the current study to evaluate the associations between cognitive performance and BPSD. Partial Least Squares (PLS) was first used to assess whether there were any associations between age, education, cognitive ability, and the NPI-Q factors. Afterwards, cluster analysis was applied to explore whether patients could be computationally placed in distinct clusters based on their NPI-Q and MoCA scores, and to examine the association between the generated clusters and the diagnosis of aMCI/DAT. The Supplementary file describes the machine learning techniques mentioned above in greater detail. The primary focus of this investigation was the relationship between neurocognitive functions and the BPSD (see regression in PLS analysis). Accordingly, we have computed the minimal required sample size using power analysis (G*Power 3.1.9.4) for a linear multiple regression. Given an effect size of 0.176 (equivalent to 15% explained variance), power = 0.8, alpha = 0.05, and three covariates, the sample size should be at least 66.

## 3. Results

### 3.1. Demographics, clinical, and reliability data

Table 1 shows the socio-demographic and clinical data of aMCI and patients with DAT. Total MoCA and TMSE scores were significantly lower in DAT as compared with aMCI participants. The NPI-Q and NPI total scores were higher in DAT than in aMCI.

**Table 1:** Demographic and clinical characteristics of participants with aMCI and DAT.

Characteristics	aMCI (*n* = 80)	DAT (*n* = 40)	*F*/*χ*^2^	df	*p*
Sex (M/F)	22/58	13/27	0.32	1	0.570
Age (years)	72.1 (7.3)	76.9 (9.0)	9.83	1/118	0.002
Education (scale 1–7)	5.7 (1.8)	5.3 (2.0)	8.50	1/118	0.004
Total MoCA	22.7 (4.2)	12.1 (6.0)	126.26	1/118	<0.001
Total TMSE	27.3 (2.3)	18.6 (5.7)	141.10	1/118	<0.001
NPI total	1.14 (3.05)	4.10 (5.94)	13.11	1/118	<0.001
NPI-Q total	0.56 (1.48)	2.45 (3.15)	19.62	1/118	<0.001
NPI severity	0.72 (1.63)	2.58 (3.16)	17.86	1/118	<0.001
NPI-Q severity	0.57 (1.48)	2.45 (3.15)	19.70	1/118	<0.001
NPI distress	0.26 (0.85)	0.97 (1.89)	8.08	1/118	0.005
NPI-Q distress	0.23 (0.76)	1.20 (2.63)	9.46	1/118	0.003

All results are shown as mean (SD) or as a ratio. Results of the analysis of variance (F) or contingency analysis (*χ*^2^ test). aMCI, amnestic Mild Cognitive Impairment; DAT, Dementia of Alzheimer’s Type; Education: ordinal score of 1: no education, 2: below primary 6, 3: primary school, 4: middle school, 5: secondary school, 6: diploma, and 7: bachelor’s degree or higher; MoCA, Montreal Cognitive Assessment; TMSE, Thai Mini-Mental State Examination; NPI, Neuropsychiatric Inventory; NPI-Q, Neuropsychiatric Inventory Questionnaire.

None of the participants reported euphoria/elation, nevertheless, internal consistency of the NPI-Q was calculated with both the inclusion and exclusion of euphoria to evaluate whether the presence of this symptom would significantly affect the measure. Cronbach’s coefficient alpha computed on all NPI-Q items in the study groups showed good internal consistency (>0.7) with and without inclusion of euphoria (Supplementary Table S1). Regarding concurrent validity (Table 2), correlations revealed that the total MoCA, NPI-Q, and NPI scores were significantly and negatively correlated in the total participant sample (aMCI + DAT) and the DAT sample (except for the correlation between total MoCA and the NPI-Q distress score), but not in aMCI alone. Additionally, there were statistically significant positive interscale correlations among the NPI and NPI-Q scores (total, severity, and distress) (Supplementary Table S2), as well as the subdomain scores of the two tests (with the exception of the agitation/aggression and dysphoria/depression domain for the NPI-Q and NPI distress scores) (Supplementary Table S3).

**Table 2:** Spearman’s rank order correlation coefficients between MoCA, NPI, and NPI-Q scores, both separately and combined.

	aMCI (*n* = 80)	DAT (*n* = 40)	aMCI and DAT (*n* = 120)
Total MoCA and total NPI-Q severity score	−0.173 (0.126)	**−0.385 (*p* < 0.014)**	**−0.395 (*p* < 0.001)**
Total MoCA and NPI-Q distress score	−0.117 (0.303)	−0.195 (0.229)	**−0.245 (*p* < 0.001)**
Total MoCA and total NPI-Q score	−0.130 (0.250)	**−0.385 (*p* < 0.001)**	**−0.383 (*p* < 0.001)**
Total MoCA and NPI severity score	−0.007 (0.951)	**−0.351 (*p* < 0.026)**	**−0.300 (*p* < 0.001)**
Total MoCA and NPI distress score	−0.022 (0.846)	**−0.315 (0.050)**	**−0.259 (*p* < 0.001)**
Total MoCA and total NPI score	−0.011 (0.919)	**−0.376 (0.017)**	**−0.294 (0.001)**

Bold numbers indicate statistical significance. MoCA, Montreal Cognitive Assessment; NPI, Neuropsychiatric Inventory; NPI-Q, Neuropsychiatric Inventory Questionnaire; aMCI, amnestic mild cognitive impairment; DAT, Dementia of Alzheimer’s Type.

The test-retest reliability of two different NPI-Q measures administered to the same individuals (10 aMCI and 10 patients with DAT) within 1 week of each other was adequate with an intra-class correlation coefficient of 0.986 (95% confidence interval: 0.965; 0.994). In patients with DAT, the intra-class correlation coefficient was 0.979 (95% confidence interval: 0.922; 0.995).

### 3.2. Exploratory factor analysis

EFA was carried out on the total sample (*n* = 120). As none of the patients reported euphoria/elation, this domain was omitted from the factor analysis. Table 3 summarizes the findings of two EFAs conducted on the 11 NPI-Q domains. The KMO score for sampling adequacy was adequate, allowing EFA to be used on our dataset. Results from the analysis indicated that the first factor explained 43.9% of the variance while the addition of a second factor improved this value to 56.5%. The Hull test, parallel analysis (PA), and the Bayesian Information Criterion (BIC) test all indicated that the recommended number of factors to be retained was one. As seen in Table 3, all 11 domains loaded strongly on this first factor. Nevertheless, the values of unidimensional congruence (UNICO) (0.882), explained common variance (ECV) (0.715), and mean of item residual absolute loadings (MIREAL) (0.307) did not suggest that the data should be regarded as principally unidimensional. Moreover, the root mean square of residuals (RMSR) values (Table 3) implies that the one factor model did not match the data well. Consequently, we investigated a bifactor and a two-factor model.

**Table 3:** Exploratory factor analysis results on the NPI-Q rating scale domains in aMCI and patients with DAT.

Domain	Factor 1	Exploratory bifactor analysis	
		General factor	Single-group factor
1. Delusions	**0.707**	**0.473**	**0.652**
2. Hallucinations	**0.481**	**–**	**0.731**
3. Agitation/aggression	**0.391**	**0.325**	**–**
4. Dysphoria/depression	**0.527**	**0.603**	**–**
5. Anxiety	**0.668**	**0.923**	**–**
6. Apathy/indifference	**0.810**	**0.687**	**0.402**
7. Disinhibition	**0.539**	**0.446**	**–**
8. Irritability	**0.752**	**0.791**	**–**
9. Aberrant motor	**0.554**	**0.539**	**–**
10. Nighttime disturbances	**0.431**	**0.339**	**–**
11. Appetite/eating disturbances	**0.829**	**0.6283**	**0.589**
Quality data			
Kaiser-Meyer-Olkin	0.76697	0.76697	
Factor determinacy index	0.950	0.965	0.904
Marginal reliability	0.831	0.930	0.817
Sensitivity ratio	2.218	3.654	2.115
Expected percentage of true differences	89.7%	94.1%	89.2%
Generalized H index	0.832	0.930	0.817
Root mean square of residuals (Kelley’s criterion)	0.1226 (0.0917)	0.0909 (0.0917)	

Exploratory factor analysis (one factor and bifactorial solutions) performed on the 11 domains of the NPI-Q rating scale. Significant loadings are shown in bold. Loadings lower than 0.3 (non-significant loadings) are omitted. NPI-Q, Neuropsychiatric Inventory Questionnaire; aMCI, amnestic mild cognitive impairment; DAT, Dementia of Alzheimer’s Type.

The results (see Table 3) suggested that the former model was adequate, while the two-factor model was not. Table 3 (right columns) summarizes the most adequate model for fitting the 11 domains in aMCI/DAT: a bidimensional oblique model with a general factor (GF) representing overall BPSD severity and a single-group factor (SGF) that highly loaded onto four domains, namely delusions, hallucinations, apathy, and eating disturbances. Both the GF and SGF had sufficient construct replicability and were successfully measured, as indicated by the Factor Determinacy index, marginal reliability, and expected percentage of true differences. Overall, a bifactor solution fitted the data well with a GF that was well characterized by all domains (except hallucinations) and a SGF that was well defined by psychotic symptoms, apathy, and appetite disorders.

### 3.3. Partial least squares structural equation modeling (PLS-SEM) path analysis

**Figure 1:**
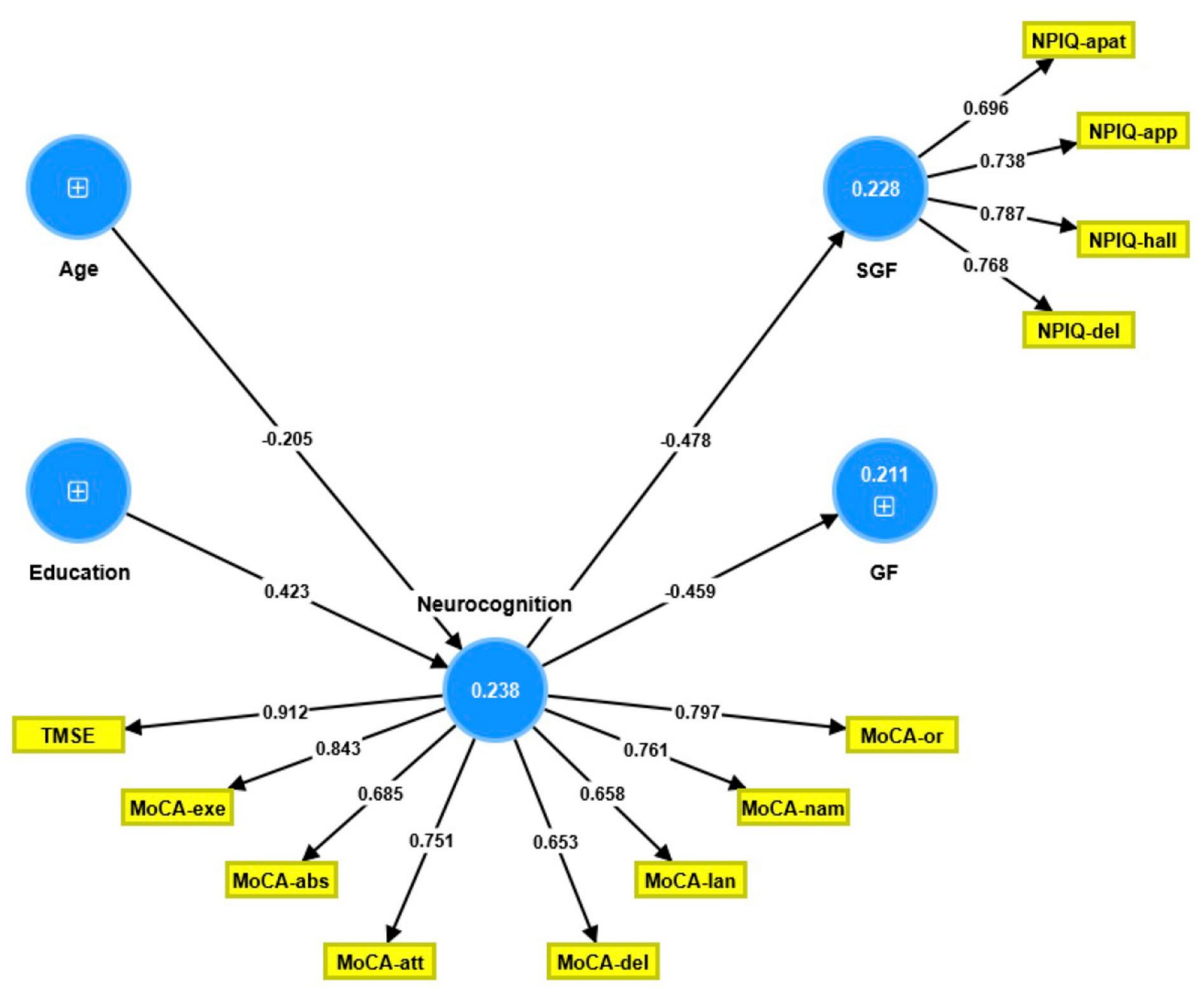
Partial least square path model. This model shows that the neurocognitive scores (conceptualized as a latent vector) predict both the general Neuropsychiatric Inventory Questionnaire (NPI-Q) factor score and the single group NPI-Q factor score. The latter was conceptualized as a factor extracted from four NPI-Q subdomains. Age and education predicted part of the variance in the neurocognitive factor score. Shown are the path coefficients (all *p* < 0.001, except age: *p* = 0.008) and the loadings on the outer models (all *p* < 0.001). The figures within the blue circles indicate explained variance. GF, general factor; SGF, single-group factor; NPIQ, Neuropsychiatric Inventory Questionnaire; apat, apathy; app, appetite; hall, hallucinations; del, delusions; TMSE, Thai Mini-Mental State Examination; MoCA, Montreal Cognitive Assessment; or, orientation; nam, naming; lan, language; del, delayed recall; att, attention; abs, abstract thinking; exe, executive functions.

The partial least squares structural equation modeling (PLS-SEM) path model shown in Figure 1 portrays the correlations between age and education (entered as input variables), cognitive dysfunctions (mediator variable), and the NPI-Q general and single-group factors (output variables). The single-group factor was introduced as a latent vector derived from four NPI-Q items: delusions, hallucinations, apathy, and appetite changes. Neurocognitive impairments were conceptualized as a factor derived from the 6 MoCA domains and the total TMSE score, while age, education, and the general NPI-Q factor were entered as single indicator variables. With SRMR = 0.060, the model provided in Figure 1 exhibits an acceptable fit.

The single-group latent vector demonstrated sufficient construct reliability, as illustrated by a composite reliability of 0.835, Cronbach’s alpha of 0.740, rho A of 0.738, and extracted average variance of 0.560. All four items loaded strongly (all >0.6) and significantly (all *p* < 0.001). The GF was entered as a single indicator. Additionally, the neurocognition latent vector demonstrated sufficient construct reliability, with a composite reliability of 0.916, Cronbach’s alpha of 0.895, rho A of 0.911, and an average variance extracted of 0.581. Both the TMSE and MoCA subdomains loaded highly (all >0.6) and substantially (all *p* < 0.001). Blindfolding demonstrated that the single-group (0.095) and neurocognition (0.134) factors had acceptable construct cross-validated redundancies. Confirmatory Tetrad Analysis established that neither vector was misspecified as reflective. We discovered that 21.1% of the variance in the GF indicator was explained by the regression on the neurocognition component and that 22.8% of the variance in the single-group latent vector was explained by the neurocognitive vector; age and education explained 23.8% of the variance in the latter. Thus, education (*t* = 4.20, *p* = 0.001) and age (*t* = 2.44, *p* = 0.015) exhibited specific indirect effects on both NPI-Q components, with the neurocognitive factor mediating these effects.

### 3.4. Cluster analysis

The two-step cluster analysis conducted on the NPI-Q domain scores, the total MoCA score, and the diagnosis in the total study sample retrieved three clusters with an accurate silhouette measure of cohesion and separation of 0.6. Table 4 shows the features of these clusters. All aMCI participants, except for 8 individuals, were allocated into Cluster 1 while all patients with DAT were separated into Clusters 2 and 3. The remaining 8 aMCI subjects were all allocated to Cluster 3. Cluster 1 was characterized by high total MoCA and low NPI-Q scores, Cluster 2 had low total MoCA and low NPI-Q scores, and Cluster 3 had low MoCA and high NPI-Q scores. Accordingly, the cluster analysis retrieved a group of subjects with high BPSD (Cluster 3: 18 DAT and 8 aMCI subjects). Supplementary Figure S1 shows the score distributions of the NPI-Q domains in the three clusters.

**Table 4:** Results of cluster analysis performed on the NPI-Q domain scores.

Variables	Cluster 1 *n* = 72	Cluster 2 *n* = 22	Cluster 3 *n* = 26	*F*/*χ*2/FFHET	df	*F*/*Χ*^2^
aMCI/DAT	72/0	0/22	8/18	112.61	–	<0.001
Total MoCA score	22.8 (4.3)	14.0 (5.3)	13.4 (7.6)	42.44	2/117	<0.001
Total NPI-Q score	0.10 (0.34)	0.14 (0.35)	5.12 (2.32)	84.25	2/117	<0.001
Age (years)	72.1 (6.9)	75.4 (9.9)	77.0 (8.9)	4.17	2/117	0.018
Male/female ratio	18/54	7/15	10/16	1.77	2	0.413
Education	5.8 (1.8)	4.9 (2.4)	4.6 (2.0)	4.22	2/117	0.017

All results are shown as mean (SD) or as a ratio. FFHET, Fisher-Freeman-Halton Exact test; aMCI, Amnestic mild cognitive impairment; DAT, Dementia of Alzheimer’s Type; MoCA, Montreal Cognitive Assessment; NPI-Q, Neuropsychiatric Inventory Questionnaire; Education: see Table 1; NS, non-significant.

## 4. Discussion

The purpose of this study was to validate the Thai NPI-Q as a good marker for BPSD and to examine the relationship between cognitive performance and BPSD in an elderly population with aMCI and DAT. With this knowledge, faster and reliable BPSD detection can be accomplished in the Thai population and more appropriate patient treatment and management can be put in place.

### 4.1. Validity of the Thai NPI-Q

The first major finding is that the Thai NPI-Q has good validity as reflected by the internal consistency, concurrent validity, interscale correlations, and test-retest reliability measures. Compared to the original English NPI-Q version (9), the interscale correlations amongst the NPI, NPI-Q, and cognitive performance metric for this current study was considerably similar in significance and value. Expanding on this, the correlation values between the NPI and NPI-Q in this study were slightly lower compared to the original authors (but still significant), while the correlation values between the NPI-Q total score and cognitive performance scores in both papers were negative and highly similar.

For the subdomain interscale correlations between the NPI and NPI-Q, most values in this current study were similar to those reported in the original NPI-Q paper (9). Notably, this study found a higher test-retest reliability than the original authors.

Taken together, the Thai NPI-Q is comparable to the NPI and is a good, quick alternative tool to measure BPSD in aMCI and patients with DAT.

### 4.2. Psychometric properties of NPI-Q

The second major finding of this study is that a general factor underpins the BPSD manifestations, as indicated by the results of the EFA. The one factor solution showed that one single factor largely explained the variance in the BPSD and, importantly, all items (except euphoria) significantly loaded onto this latent construct. This implies that all NPI-Q severity items are manifestations of a shared common core, namely severity of BPSD (29). However, as the root mean square of residuals was not satisfactory, we improved the factor solution by examining a pure exploratory bifactor analysis. We found a general factor (loading on all items except hallucinations) and a single-group factor (loading on delusions, hallucinations, apathy, and appetite) underpinning the NPI-Q domains. Our results of a bifactor structure for the NPI-Q was notably different from another study on patients with DAT that found a two-factor structure consisting of Negative/Oppositional and Anxiety/Restlessness factors (30). Other studies performed on the NPI and NPI-Q reported between 3 and 5 factors (31–35). The discrepancies in the findings could potentially be due to language or cultural differences that cause BPSD to manifest differently. The bifactorial structure found underlying the NPI-Q in this study confirms that a common core underpins all test items, appropriately reflecting the severity of BPSD, and that the sum of all items is a good index representing psychopathology severity. Furthermore, the single-group dimension suggests that increased overall BPSD severity is accompanied by the emergence of single-group symptoms.

### 4.3. Relationship between cognitive performance and NPI-Q

The third major finding is that a general decline in neurocognitive functions (as reflected by a latent vector extracted from the different MoCA domains) predicts the severity of the general and single-group NPI-Q factors. To the best of our knowledge, existing literature thoroughly examining the relationship between cognitive performance and BPSD is limited, and none have assessed cognitive performance using the MoCA (36–38). Therefore, there is difficulty in making detailed comparisons between cognitive test subdomains. The observed significant negative correlation between the total MoCA, NPI-Q, and NPI (total and severity) scores in patients with DAT in this study is in line with the overall agreement that BPSD are common in dementia, regardless of dementia type (35), and that neuropsychiatric symptoms (e.g., activity disturbances and agitation) progress in parallel with severity of cognitive decline (36). These findings extend those of a previous study that stated that impairments in verbal fluency, executive functions, and episodic and semantic memory were associated with severity of neuropsychiatric symptoms (37).

Regarding cognitive deficits in aMCI individuals, the presence of executive dysfunction and other specific neurocognitive deficits were reported to be predictors of greater BPSD symptom severity in these individuals, particularly depression and anxiety (39). However, the current study did not observe such associations possibly because the deficits were less severe and not all cognitive domains were affected in these individuals.

The PLS results indicate that around 21–22% of the variance in both general and single-group factors may be explained by cognitive deficits, suggesting that a large part of the variance in BPSD remains unexplained. Genetic and psycho-social factors and adverse outcome pathways may further explain the onset of BPSD (35).

Importantly, from our two-step cluster analysis, 3 clusters were discovered: one cluster with normal total MoCA and NPI-Q scores, a second cluster with only patients with DAT with low MoCA but normal NPI-Q scores, and a third cluster with low MoCA and high NPI-Q scores. Cluster 3 comprised of 18 DAT and 8 aMCI patients, suggesting that aMCI subjects with high NPI-Q scores were allocated to a DAT subgroup. According to the clustering, over 50% of our patients with DAT suffered from BPSD, whereas only 10% of aMCI patients exhibited BPSD. There is currently limited information on the natural course of BPSD in aMCI. It has been reported that a significant percentage of aMCI individuals have at least one persistent symptom (12), though there has been no mention of MCI patients with severe symptoms in all neuropsychiatric domains. Previous papers showed that up to 90% of patients with DAT may suffer from BPSD and that BPSD scores are associated with a poor outcome (35). Therefore, it is plausible that aMCI subjects with severe NPI-Q symptoms (10% of our sample) may have a poorer prognosis and constitute a subgroup of aMCI patients who will likely convert into probable DAT. This suggests the potential use of BPSD as a predictor of dementia severity.

### 4.4. Limitations

First, all patients were recruited from the same tertiary care hospital, and hence the findings may not be fully generalizable to the entire Thai population. Second, future studies should collect additional data from more diverse locations and examine whether psychotropic drugs, including antidepressants, may improve BPSD. Third, some studies have reported differences in BPSD frequency between early and late onset forms of AD (40–42), therefore, future research should explore the effect of onset on cognitive performance.

## 5. Conclusion

This study demonstrates that the Thai NPI-Q test is of satisfactory quality and accurately assesses BPSD, making it comparable to the Thai NPI test. The neurocognitive deficits observed in DAT, which are absent in aMCI, can predict the general and single-group factors that underpin the BPSD assessed using the NPI-Q.

## Data availability statement

The original contributions presented in the study are included in the article/Supplementary material, further inquiries can be directed to the corresponding author.

## Ethics statement

The studies involving human participants were reviewed and approved by IRB Committee, Faculty of Medicine, Chulalongkorn University. The patients/participants provided their written informed consent to participate in this study.

## Author contributions

SH, ST, TC, MP, PC, PM, YL, KP, RA, AA, and MM contributed to this manuscript. Statistical analyses were performed by MM. All authors contributed to the article and approved the submitted version.

## Funding

This research was supported by Chulalongkorn University. The sponsor had no role in the data or manuscript preparation.

## Conflict of interest

The authors declare that the research was conducted in the absence of any commercial or financial relationships that could be construed as a potential conflict of interest.

## Publisher’s note

All claims expressed in this article are solely those of the authors and do not necessarily represent those of their affiliated organizations, or those of the publisher, the editors and the reviewers. Any product that may be evaluated in this article, or claim that may be made by its manufacturer, is not guaranteed or endorsed by the publisher.
