# Supplementary material for: Cognitive impairments predict the behavioral and psychological symptoms of dementia
Source: Front Neurol. 2023 Jul 20;14:1194917. doi: 10.3389/fneur.2023.1194917 (PMC10400323; doi:10.3389/fneur.2023.1194917)
Supplement: Supplementary file 1 [file Data_Sheet_1.pdf]

Electronic Supplementary File (ESF)

1 Machine Learning techniques

1.1 Exploratory Factor Aanalysis

We used exploratory factor analysis (EFA) as a data-driven approach to study the component structure of the NPI-Q in aMCI/AD. To determine the dataset's factor structure, EFA was performed using FACTOR, version 10.5.03 for Windows[1,2]. To extract factors, a robust unweighted least squares (RULS) technique was utilized using 500 bootstrap samples[1-3]. The dispersion matrix was constructed using Pearson's and polychoric correlations, and the analysis was robust using bias-corrected and accelerated (Bca) bootstraps. Different models were examined including one or more factor models and pure EFA Bifactor Model with Promin rotation. Prior to doing EFA, the Kaiser-Meyer-Olkin (KMO) index was employed to determine if the matrix was suitable for factorization. Schwartz's Bayesian Information Criterion (BIC), the Hull test, and Parallel Analysis (Optimal Implementation) were used to determine the dimensionality and estimate the number of factors to be retained. The degree of unidimensionality was determined using unidimensional congruence (UNICO), explained common variance (ECV), and the mean of item residual absolute loadings (MIREAL). When UNICO is more than 0.95, ECV is greater than 0.85 and MIREAL is greater than 0.300 – the data should be regarded as basically unidimensional. The root mean square of residuals (RMSR), with an expected mean value of RMSR for an appropriate model (Kelley's criterion), was used to assess the residual distribution. The H index was used to assess construct replicability with values greater than 0.80 suggesting high latent vector replicability and consistency across studies. The factor determinacy index (FDI), marginal reliability, and expected proportion of true differences were used to assess the quality of factor score estimates.

Partial Least Squares (PLS) analysis

The causal associations between age, education, neuroconitive deficits, and the NPI-Q factors were assessed using the SmartPLS path analysis approach (SmartPLS version 3.2.3)[4]. Each variable was input as a single indicator or as a latent vector extracted from its reflective manifestations. When the outer and inner models met predefined quality criteria, complete PLS analysis was performed namely a) the model fit SRMR is less than 0.08; b) all latent vectors have a high composite reliability (> 0.7), Cronbach's alpha (> 0.7), and rho A (> 0.8), with an average variance extracted greater than 0.5; and c) all loadings on the latent vectors are greater than 0.6 at $p < 0.001$. Moreover, these models were not mis-specified as reflective models as tested with Confirmatory Tetrad Analysis. Complete PLS analysis using 5,000 bootstrap samples was performed to compute specific indirect, total indirect, and total direct path coefficients (with exact *p*-values).

Cluster analysis

We used two-step cluster analysis to discover clusters of patients based on the NPI-Q scores. Two-step cluster analysis was performed using IBM SPSS windows version 28.

ESF Table 1 Internal consistency of the NPI-Q in participants as assessed using Cronbach's coefficient alpha

Patient Groups	With Euphoria	Without Euphoria
**Amnestic Mild Cognitive Impairment**	0.747	0.760
**Alzheimer's Disease**	0.775	0.783
**Total**	0.796	0.804

NPI-Q: Neuropsychiatric Inventory Questionnaire.

ESF Table 2 Interscale Spearman rank order correlation coefficients between NPI and NPI-Q scores in participants

Variables	Amnestic Mild Cognitive Impairment (n=80)	Alzheimer's Disease (n=40)	Total (n=120) (*p-value*)
**Total NPI-Q and total NPI severity scores**	0.690 ($p<0.001$)	0.976 ($p<0.001$)	0.867 ($p<0.001$)
**Total NPI-Q and NPI score**	0.705 ($p<0.001$)	0.972 ($p<0.001$)	0.867 ($p<0.001$)
**Total NPI-Q and NPI distress score**	0.737 ($p<0.001$)	0.799 ($p<0.001$)	0.782 ($p<0.001$)

This table shows the interscale correlations among the Neuropsychiatric Inventory (NPI) and Neuropsychiatric Inventory Questionnaire (NPI-Q) scores (total, severity, and distress). There was a statistically significant positive correlation between the total NPI-Q and NPI score, severity, and distress scores in the total study group, patients with Alzheimer's Disease (AD), and patients with amnestic mild cognitive impairment (aMCI).

ESF Table 3 Interscale correlation coefficients between each individual domain of the Neuropsychiatric Inventory (NPI) and Neuropsychiatric Inventory-Questionnaire (NPI-Q) rating scales (n=120)

Domain	NPI-Q and NPI severity scores	NPI-Q and NPI distress Scores	Total NPI-Q and NPI scores
**1. Delusions**	0.960*	0.901*	0.979*
**2. Hallucinations**	0.960*	0.912*	0.941*
**3. Agitation/Aggression**	0.762*	NS	0.721*
**4. Dysphoria/Depression**	0.879*	.NS	0.763*
**5. Anxiety**	0.823*	0.462*	0.715*
**6. Apathy/Indifference**	0.749*	0.314*	0.672*
**7. Disinhibition**	0.861*	0.538*	0.738*
**8. Irritability/Lability**	0.797*	0.731*	0.677*
**9. Aberrant Motor**	0.801*	0.674*	0.806*
**10. Nighttime Disturbances**	0.670*	0.437*	0.569*
**11. Appetite/Eating Disturbances**	0.902*	0.893*	0.805*

* All significant at $p<0.001$; NS: non-significant. Analysis of the interscale correlations between the individual domains of the NPI and NPI-Q indicate that the correlations between all domains of the two tests were statistically significant at the <0.001 level except for agitation/aggression domain and dysphoria/depression domain.

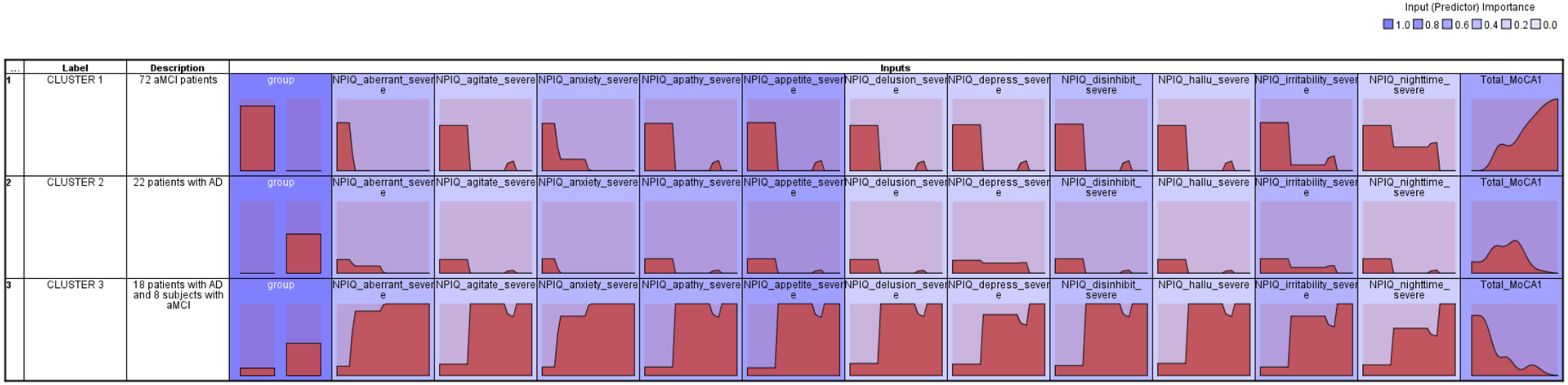

ESF Figure 1. Results of cluster analysis showing the score distributions of the NPI-Q domains and the MoCA in the three clusters. *NPI-Q: Neuropsychiatric Inventory Questionnaire; MoCA: Montreal Cognitive Assessment*

References

1 Ferrando PJ, Lorenzo-Seva, U. *Unrestricted item factor analysis and some relations with item response theory*. https://psico.fcep.urv.cat/utilitats/factor/documentation/technicalreport.pdf [Accessed 20th November 2022].

2 Ferrando PJ, Lorenzo-Seva U. Program FACTOR at 10: Origins, development and future directions. *Psicothema*. 2017;29(2):236-40.

3 Lloret S, Ferreres A, Hernández A, Tomás I. El análisis factorial Exploratorio de los ítems: Análisis Guiado según Los Datos empíricos y el software. *Anales de Psicología*. 2017;33(2):417.

4 Ringle CM, Wende S, Becker, JM. *SmartPLS 3*. Version 3.2.3. Boenningstedt, DE: SmartPLS GmbH; 2015.
